# Effectiveness of WHO-recommended antenatal care visits, ultrasonography, and health education in reducing unnecessary caesarean sections among pregnant women in Bangladesh: a hospital-based randomised controlled trial

**DOI:** 10.7189/jogh.15.04182

**Published:** 2025-06-27

**Authors:** Habiba Shirin, Huq K A T M Ehsanul, Mohammad Delwer Hossain Hawlader, Sumaya Binte Masud, Kamrunnahar Misty, Farhana Dewan, Michiko Moriyama

**Affiliations:** 1 Graduate School of Biomedical and Health Sciences, Hiroshima University, Hiroshima, Japan; 2 Department of Public Health, North South University, Dhaka, Bangladesh; 3 Public Health Promotion and Development Society (PPDS), Dhaka, Bangladesh; 4 Obstetrical and Gynaecological Society of Bangladesh (OGSB), Dhaka, Bangladesh

## Abstract

**Background:**

The rate of caesarean sections (C-sections) is increasing globally, with many procedures being performed without clinical indication. We aimed to explore whether implementing the eight antenatal care visits that include health education and four ultrasonographic examinations (intervention group (IG)) recommended by the World Health Organization (WHO) would reduce unnecessary C-sections among Bangladeshi pregnant women in comparison to the usual four antenatal care and two ultrasonogram exams (comparison group (CG)) currently in use within the country’s healthcare system.

**Methods:**

We designed a randomised controlled trial that included two tertiary and two non-tertiary hospitals in Bangladesh. The randomisation was done at the hospital level, whereby we randomly allocated the hospitals to the IG or CG, with one tertiary and one non-tertiary hospital in each arm. We conducted this trial between November 2021 and December 2022. Pregnant women of all ages without any complications requiring C-sections were enrolled in the allocated IG and CG hospitals. The primary outcome was the reduction in the number of unnecessary C-sections. We considered unnecessary C-sections when there was no proper indication for caesarean delivery. Alongside descriptive analysis, we used adjusted log-linear regression models to investigate the relationship between unnecessary C-sections and various contributing factors.

**Results:**

We enrolled 288 pregnant women from two intervention and two comparison hospitals, with 144 assigned to the IG and 144 to the CG (72 from each hospital). There were 183 complete, successful deliveries in total (50.27% in IG *vs*. 49.73% in CG; *P* = 0.903). Of these, 106 resulted in C-sections (39.62% in IG and 60.38% in CG; *P* = 0.001), with 70 assessed as being necessary (47.14% in IG and 52.86% in CG; *P* = 0.027) and 36 as being unnecessary (25% in IG and 75% in CG). There were significantly fewer unnecessary C-sections among the women in the IG (*P* = 0.027), who also had a significantly lower risk ratio of 0.64 (95% confidence interval = 0.54–0.77, *P* < 0.001) compared to the CG.

**Conclusions:**

Our findings indicate that the WHO-recommended antenatal visits and their interventions were effective in reducing unnecessary C-sections by 50%.

**Registration:**

Clinicaltrials.gov: NCT05135026.

Since 1985, the World Health Organization (WHO) has held the estimate that the ideal rate of caesarean sections (C-sections) to range between 10.0% and 15.0% of all deliveries [1]. Yet at the global level, C-sections rose from 7% in 1990 to 21% in 2021, with significant discrepancies in rates of access observed in 2021, varying from the highest of 43% in Latin America and the Caribbean to the lowest of 5% in sub-Saharan Africa [2]. This trend is likely to increase over the next decade, with C-sections expected to account for nearly 29% of all births by 2030 [2].

The indications for a C-section include prolonged or obstructed labour, foetal distress, and abnormal presentation of the baby [2]. Unfortunately, most of the elective C-sections were performed at mothers’ request, either due to their fear of vaginal delivery or them having a uterine scar from the previous delivery [3]. While they are lifesaving interventions, C-sections are also associated with increased maternal and perinatal morbidity [4,5] due to short- and long-term risks affecting the health of women and children and future pregnancies. In addition to higher healthcare costs, a C-section is associated with uterine rupture, ectopic pregnancy, abnormal placentation, stillbirth, and preterm birth [4]. Children born by C-section may also experience various physical, hormonal, and bacterial exposures, as well as short-term complications such as altered immune development, allergy, atopy and asthma, and reduced intestinal gut microbiome diversity [5]. The WHO recommends health educational intervention and more frequent ultrasonographic examinations to reduce medically unnecessary C-sections [2].

C-section rates continue to increase in Bangladesh, rising from 33% in 2017 to 45% in 2022 [6]. Medically unwarranted C-sections were estimated to account for 77.1% of all C-sections in the country in 2018, representing an increase of 51% from 2016 [7]. In 2016, the WHO antenatal care (ANC) model included performing an ultrasonography (USG) examination before 24 weeks of gestation during pregnant women’s first contact up to 12 weeks or the second contact at 20 weeks of gestation. This early USG facilitates estimating the gestational age, improves detection of foetal anomalies and multiple pregnancies, reduces the induction of labour in post-term pregnancy, and improves a woman's pregnancy experience. If it is not performed before 24 weeks, a late USG could be considered to identify presentation, the position of the placenta, and the number of foetuses [8]. In Bangladesh, the compliance with the required provision of USG in healthcare centres was <50%, while the community clinics with ANC facilities did not have available USG services [9].

Health education significantly improves patients’ understanding of medical information, thereby increasing their utilisation of treatment facilities and their medication adherence, and consequently improving their clinical outcomes [10–12]. One study observed a higher number of C-sections among pregnant women who did not receive prenatal health education [13], which would suggest that ANC-related health education could be crucial in increasing awareness and preventing unnecessary C-sections [14]. In Bangladesh, 72% of females aged ≥15 years were reported to be literate in 2020 [15], which is concerning in this context, given that those with secondary and higher education achieved high quality of ANC [16] and received more USG than their uneducated counterparts [17].

In 2016, the WHO increased the number of recommended ANC visits to healthcare providers from 4 to 8, as they could potentially reduce perinatal death by 8 per 1000 live births [18]. This new standard care of ANC visits has been shown to reduce maternal mortality among all pregnant women, including adolescent girls and those living in hard-to-reach areas and conflict zones [18,19]. Globally, however, only 64% of pregnant women receive four or more ANC care visits during their pregnancy [18]. Aside from reducing perinatal deaths, the increased standard ANC visits helped decrease the pregnant women’s anxiety due to the shorter time gap between the visits [20]. The increased facility-based antenatal contacts could also improve safety during pregnancy by way of detecting undetected abnormalities in pregnant women and help assess foetal conditions better [19]. A greater number of ANC contacts with knowledgeable, respectful, and supportive health practitioners also helped improve the satisfaction of pregnant women and their families with the care provided [19]. Despite this, Bangladesh continues to recommend four ANC visits and two USGs throughout the country, in contrast to the WHO recommendation; more importantly, while the proportion of pregnant women with at least one ANC visit increased from 82% to 88% between 2017 and 2022, the rates of four or more ANC visits reduced from 46% to 41% [6]. 

Few studies have focussed on the effectiveness of combining interventions to reduce unnecessary C-sections (**Box 1**). Therefore, we aimed to determine if combining health education, additional USGs, and the WHO-recommended eight ANC visits would be effective in reducing unnecessary C-sections among pregnant women in Bangladesh.

Box 1 **Research in context**

**Evidence before this study**

We systematically reviewed the scientific literature through PubMed and Google Scholar to identify all articles published in English before 20 March 2024 by using a combination of the following keywords: caesarean section, unnecessary C-section, ultrasonography in pregnancy, health education and ANC. We found that several intervention studies reported reductions in C-sections, but that very few (n = 23) were conducted to reduce unnecessary C-sections. These 23 studies provided a single intervention for reducing unnecessary C-sections, meaning that none combined multiple interventions such as health education, additional USGs, and the WHO-recommended eight ANC visits.

**Added value of this study**

We designed a trial with a hospital-based randomisation of selected tertiary and non-tertiary hospitals in Bangladesh. We provided health education, additional USGs, and the WHO-recommended eight ANC visits to identify pregnancy-related risk factors in their early pregnancy to enable their families to prepare for safe delivery and prevent unnecessary C-sections.

**Implications of all the available information**

In the context of inadequate knowledge about ANC visits and the high rates of unnecessary C-sections in low- and middle-income countries, our findings suggest that a safe and convenient intervention – *i.e.* the WHO-recommended eight ANC visits supplemented by health education and USGs, can be an effective intervention for pregnant women.

## METHODS

### Study design

This was an open-label randomised controlled clinical trial (RCT). The randomisation was performed at the hospital level with an equal stratification of tertiary and non-tertiary hospitals (one tertiary and one non-tertiary each to the intervention group (IG) and control group (CG)) to minimise the risk of selection bias and to prevent participant contamination. The aim was to assess the effectiveness of health education, additional USGs, and the WHO-recommended eight ANC visits in reducing unnecessary C-sections in Bangladeshi tertiary and non-tertiary hospitals. We focussed on these two types of hospitals to obtain representative samples, as they are the main centres for providing maternal and child health services in Bangladesh. For this reason, we explored the differences in the implementation of these interventions between these two settings and compared the health outcomes, morbidity, and mortality among their users [21]. Recruitment of participants started in November 2021, and follow-up was completed in December 2022.

### Tertiary hospital setting

**Figure 1:**
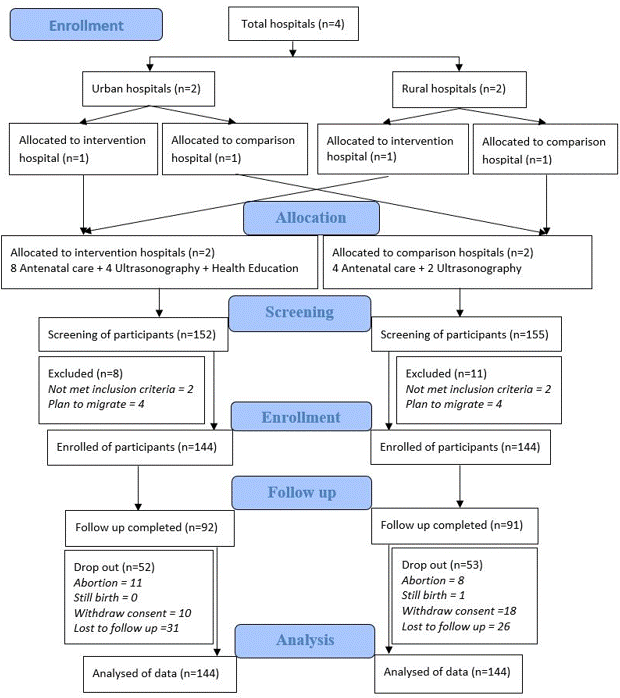
CONSORT diagram displaying distribution of hospitals between the study arms, exclusion criteria, and results of follow-up.

For reasons of accessibility, we purposefully selected two tertiary urban public health facilities, the Dhaka Medical College Hospital (DMCH) and the Sir Salimullah Medical College Mitford Hospital (SSMC), both located in Dhaka, the capital city of Bangladesh (**Figure 1**). The DMCH deals with an average of 20–25 ANC patients and admits around 50–60 obstetrics patients daily. It has a family planning clinic, a routine and a high-risk ANC corner, and a post-natal care clinic. The SSMC treats around 30–50 ANC patients and admits 50–60 obstetric cases daily. The average C-section rate at both hospitals was 53.38% in 2018 [22]. Both facilities are well-equipped to manage gynaecological and obstetrical patients. Using the randomizer.org online software [23], we randomly selected SSMC for the intervention and DMCH for the comparison hospital by using a computer-generated randomisation technique, set up by a member of the research staff who was not involved in our study. As the randomisation has been done at the hospital level, there was no need for concealment for the participants or the researchers.

### Non-tertiary hospital setting

In a similar way (**Figure 1**), we purposefully selected two non-tertiary (district level) facilities: Munshiganj District Hospital (MDH) and Bogra Mohammad Ali District Hospital (BDH). Both have 250 inpatient beds, as well as ANC corners and facilities for dealing with outpatient and inpatient gynaecological and obstetric facilities. The C-section rates in MDH and BDH were 41.43% and 73.93%, respectively, in 2023 (personal communication with hospital staff). We randomly assigned MDH for intervention and BDH as a comparison hospital using randomizer.org [23]. The process of study site selection and randomisation have been detailed elsewhere [24].

### Inclusion criteria

After we obtained their written informed consent, we enrolled all pregnant women, irrespective of their age, who visited the designated hospitals in their first trimester, had/had no apparent complications to influence delivery outcomes.

### Exclusion criteria

We excluded pregnant women not willing to participate or those who had an indication for C-section (co-morbidities and/or history of previous C-section) at their first trimester.

### Operational definition of terms

We defined an unnecessary C-section as one where a pregnant woman demanded the physician for delivery without any justifiable indication [25]. Planned or elective C-sections were understood as cases where the medical indication may be present or not. The planned C-section had to be indicated primarily for malpresentation, previous uterine scar, narrow pelvis, or twin pregnancies with the first twin in a breech presentation. Without any medical indication, C-sections were categorised as unnecessary [25]. Emergency C-sections were defined as cases where a C-section needed to be done very quickly for proper medical indication due to the emergency health issue of the mother and/or baby [25].

### Case management

Midwives identified pregnant women attending the outpatient antenatal corner of the study hospitals for their ANC check-ups. Those with a history of missed menstrual periods for more than a month and a half were advised to perform a pregnancy test by using a pregnancy test kit provided by the hospital. The midwives then assessed the women thus identified as pregnant and determined their eligibility to participate in the study per our criteria after informing them about the study and its purpose, as well as obtaining their written informed consent if they were willing to participate. After recruitment, the midwives conducted face-to-face interviews with the participants and filled out the study questionnaires.

All the research investigators, midwives, and laboratory staff who were directly involved in the study procedures completed the ‘Research Ethics Online Training’ course supported by the WHO to ensure they were familiar with the procedures required for the protection of research participants. The midwives additionally received protocol-specific training from the investigators before the initiation of the study. They also received monthly refresher training throughout the study period *via* a lecture by the principal investigator on how to obtain informed consent, fill up the questionnaires, and provide health education and anthropometric data collection to ensure consistency and adherence to the study protocols. This online training lasted about one hour per session and was conducted in different five sessions based on the protocol.

Participants in the IG received the research intervention in addition to the usual care, while the CG received only the usual care. Midwives checked the delivery history and documented the delivery type as necessary or unnecessary. We did not include gynaecologists and obstetricians in our study to minimise delivery decision bias. We relied on the pregnant women’s discharge sheets for their delivery information across all four hospitals. These sheets contained the indication of C-section, labelled as necessary (had complication) or as done per the mother’s request. If there was no indication of a C-section in the discharge sheet and the mother had no information about the indication of a C-section, then we considered the C-section as unnecessary. The questionnaires were sent from the hospitals to a designated research room on a weekly basis. After receiving the questionnaires, a research officer (part of study team) entered the data into a secured computer and deidentified them before analysis. Data were checked regularly by the investigators for its completeness and consistency. In case of any missing value or discrepancy, we corrected the data by using the source documents. We reported our findings per the CONSORT checklist (Checklist S1 in the **Online Supplementary Document**).

### Comparison hospitals (CG)

Study participants in the comparison hospitals received only the usual care, meaning they did not receive health education and additional USG. Although the WHO recommended eight ANC visits that include health education and more USGs to guide care for pregnant women [18], Bangladesh country guidelines still promote and practice four ANC visits and two USGs [6]. Therefore, the pregnant women in this group received the usual 4 ANC visits at 8–12, 24–26, 34, and 38 weeks of gestational age and one postnatal care 4–6 weeks following delivery. They received the usual two USGs at their first and fourth ANC visits. Standard laboratory investigations were performed and the women received pregnancy-related medications. During the interview, midwives collected sociodemographic data (*i.e.* age, education, occupation, number of household members, number of children, daily meal intake, smoking and alcohol habits and so on) and gynaecological and obstetrics history (*i.e.* abortion, stillbirth, gestational diabetes, and so on), and they performed clinical examination (*i.e.* body mass index; BMI, mid-upper arm circumference and blood pressure) and laboratory tests (*i.e.* blood for grouping and routine examination, USGs and urine for routine examination).

### Intervention hospitals (IG)

Study participants attending at the outpatient antenatal corner of the study hospitals for their first ANC visit were requested to ensure 8 ANC visits at 8–12, 20, 24–26, 30, 34, 36, 38, and 40 weeks of their gestational age and a postnatal visit after 4–6 weeks of delivery. They also received health education at each of their eight ANC visits, and two additional USG examinations at their third and fifth ANC visits. During the interview, midwives collected sociodemographic data (*i.e.* age, education, occupation, number of household members, number of children, daily meal intake, smoking and alcohol habits and so on) and gynaecological and obstetrics history (*i.e.* abortion, stillbirth, gestational diabetes, and so on), and performed clinical examination (*i.e.* body mass index; BMI, mid-upper arm circumference and blood pressure) and laboratory tests (*i.e.* blood for grouping and routine examination, USGs and urine for routine examination). They also received physical examinations, laboratory tests, and medication for pregnancy-related management at each of their ANC visits.

### Health education materials for intervention hospitals

We developed health education materials in the local language (Bengali) to increase study participants’ pregnancy-related awareness and empower them to make their own decisions to improve pregnancy outcomes. We first reviewed existing health educational materials in use for pregnant women at their hospital ANC visits and included those relevant to our study after assessing whether they fulfilled our study outcomes. We used coloured pictures and charts in the education modules and wrote the accompanying text in the native Bengali language for easy understanding. We included information related to healthy behaviour, healthcare utilisation patterns, pregnancy, and delivery-related complications and consequences. Healthy behaviour included regular intake of nutritious food; additional energy requirements during pregnancy and the benefits of healthy food for pregnant women and their upcoming offsprings; the consequences of poor nutrition; the importance of maintaining personal hygiene (including hand washing); the importance of adequate rest and sleep (how many hours per day and good sleep); intake iron and folate; appropriate immunisation; the importance and benefits of exclusive breastfeeding; saving money to make ANC visits and meet hospital expenditures including cost related to delivery, and cost for hiring an appropriate vehicle to attend the hospital for delivery. The education materials also stressed the benefits of regular and complete ANC visits, danger signs during pregnancy, the importance of performing USGs, the advantage of normal delivery, and the risk of unnecessary C-sections. We used a pictorial flip chart showing danger signs and potential risks for unnecessary caesarean delivery to increase awareness of safe delivery. The education materials further emphasised the benefits of hospital delivery with skilled birth attendance to prevent unwanted complications such as stillbirth and the importance of post-natal care for mother and baby. Hospital midwives provided face-to-face health education in their local (Bengali) language at the outpatient ANC corner during each of the eight ANC visits. They required 30–60 minutes to administer the questionnaires in a meaningful way. All the investigators periodically monitored and assessed the staff and their data collection procedures to ensure the proper execution of the study.

The Institutional Review Board of North South University formed an independent Data Safety and Monitoring Board for this study to oversee and monitor the study activities to ensure the safety of the participants and the integrity of the data. This Board was formed by two professional members who were not involved in this study activities. They convened every six months to discuss the study progress and any ethical issues such as deviations from study protocol, misconduct, and serious adverse events.

### Outcome

The primary outcome was unnecessary C-sections among pregnant women at the end of successful live deliveries.

The secondary outcomes were ANC and postnatal care visits, USG uses, institutional delivery (hospital and clinics), and delivery-related complications (antepartum and postpartum haemorrhage) and stillbirths.

### Sample size calculation

We calculated our sample size using Sta, version 15 (StataCorp LLC, College Station, Texas, USA) based on the estimated proportions of unnecessary C-sections in Bangladesh in 2018 [7]. Specifically, given that the prevalence of unnecessary C-section was reported to be as high as 77% in Bangladesh, we hypothesised a 20% reduction in the IG (20% of 0.77 = 0.154) compared to the CG, bringing the prevalence of C-sections down to 61%. Using a 5% level of significance (alpha) and an intra-cluster correlation coefficient of 0.00164, we estimated that a total of 288 participants (including a 10% attrition rate) across four hospitals would provide 80% power for detecting a significant effect. Each hospital was expected to recruit a minimum of 72 participants [26].

### Statistical analysis

We performed both intention-to-treat and per-protocol analysis. We first descriptively summarised the baseline variables of the IG and CG, comparing them using chi-squared test (for categorical variables). To assess the distribution of our data, we performed normality test and plotted a histogram with a density plot. For continuous variables, we compared normally distributed data using Student’s *t*-test and non-normally distributed data using the Mann-Whitney U test. We considered a *P*-value <0.05 as statistically significant.

As our dependent variable was dichotomous, we conducted log-linear binomial regression to evaluate the risk ratios (RRs) and its 95% confidence intervals (CIs) associated with the differences between groups. We adjusted this analysis for potential confounders to ensure that the observed associations were not influenced by extraneous variables. We additionally examined interaction effects among major factors and none were statistically significant, thereby confirming the validity of the additive assumption. We selected the covariates based on the literature review and incorporated them in the model for bivariate analysis which were adjusted accordingly in the log-linear binomial regression models. As for missing data, we performed no imputation. 

Besides Stata (mentioned above), we used SPSS, version 25.0 (IBM Corp., Armonk, New York, USA) for data analysis. The study protocol is provided in the **Online Supplementary Document**.

### Role of the funding source

This research was funded by a research grant from the Hiroshima University. The funder had no role in study design, participant recruitment, data collection, data analysis, data interpretation, or writing of the report.

## RESULTS

### Participant characteristics

A total of 307 pregnant women were screened – 152 from the intervention and 155 from the comparison hospitals. Nineteen (IG = 8, CG = 11) were excluded, as they did not meet the study eligibility criteria. In total, 288 pregnant women were enrolled from 4 different hospitals – 72 from each hospital, *i.e.* 144 in the IG and 144 in the CG group. Ninety-two participants in the IG and 91 in the CG completed their follow-ups, meaning 52 and 53 dropped out the two groups, respectively (**Figure 1**; Tables S1 and S2 in the **Online Supplementary Document**).

The overall mean age of the women was 24.31 (standard deviation (SD) = 5.07) years with a minimum and maximum of 15–39 years. Compared to the CG, the IG had significantly more participants with who were Muslim (97.92% *vs*. 92.36%), higher average number of household members (mean (x̄) =  4.50, SD = 1.77 *vs*. x̄ = 4.00, SD = 1.55), more cases with a history of hypertension (5.56% *vs*. 0.69%), more cases with a history of abortion (22.92 *vs*. 13.89%), more individuals who consumed less than three daily meals (11.11% *vs*. 5.56%) or three daily meals (78.47% *vs*. 54.17%), higher individuals on average (x̄ = 153.01 cm, SD = 6.19 *vs*. x̄ = 152.54 cm, SD = 6.98), more individuals who were underweight (15.28% *vs*. 4.17%) and healthy weight (55.56% *vs*. 44.44%), and more individuals with elevated BP (26.39% *vs*. 9.72%). In contrast, women in the CG had significantly higher age (x̄ = 25.11 years, SD = 5.05 *vs*. x̄ = 23.54 years, SD = 5.00), longer school education (x̄ = 9.99 years, SD = 3.96 *vs*. x̄ = 8.66 years, SD = 3.93), higher proportion of Hindu believers (7.64% *vs*. 2.08%), more smokers (10.42% *vs*. 1.39%), higher body weight (x̄ = 56.29 kg, SD = 9.54 *vs*. x̄ = 52.46 kg, SD = 9.86), husband’s longer school education (x̄ = 11.33 years, SD = 10.82 *vs*. x̄ = 7.91 years, SD = 4.49), higher household income (x̄ = BDT 21 212.00, SD = 9641.00 *vs*. x̄ = 19 668, SD = 12 048) and pedal swelling (3.47% *vs*. 0.00%) than those in the IG (**Table 1**). As many baseline variables differed significantly between the groups, we analysed the baseline characteristics based on the location of the hospital (Table S3 in the **Online Supplementary Document**). We also analysed the baseline characteristic based on C-section necessity (Table S4 in the **Online Supplementary Document**).

**Table 1:** Sociodemographic and clinical characteristics among the intervention and comparison groups (n = 288)

	Total (n = 288)	Intervention hospitals (n = 144)	Comparison hospitals (n = 144)	*P*-value†
**Demographic characteristics**				
Age in years, mean (SD)	24.31 (5.07)	23.54 (5.00)	25.11 (5.05)	0.004
Maternal education (years of schooling), mean (SD)	9.34 (3.97)	8.66 (3.93)	9.99 (3.96)	0.004
Religion				
*Muslim*	274 (95.14)	141 (97.92)	133 (92.36)	0.028
*Hindu*	14 (4.86)	3 (2.08)	11 (7.64)	
Mother’s occupation				
*Housewife*	270 (93.75)	135 (93.75)	135 (93.75)	1.000
*Other (formal and informal)*	18 (6.25)	9 (6.25)	9 (6.25)	
Husband’s age in years, mean (SD)	31.22 (6.09)	30.77 (6.04)	31.66 (6.13)	0.258
Husband’s education (years of schooling), mean (SD)	9.64 (8.49)	7.91 (4.49)	11.33 (10.82)	<0.001
Husband’s occupation				
*Industry worker, office non-executive, petty business*	95 (32.99)	49 (34.03)	46 (31.94)	0.129
*Skilled, office-executive, big business, overseas employment*	129 (44.79)	57 (39.58)	72 (50.00)	
*Others*	64 (22.22)	38 (26.39)	26 (18.06)	
Income in USD/mo, mean (SD)	173 (92)	168 (103)	181 (82)	0.006
Expenses in USD/mo, mean (SD)	135 (63)	136 (79)	135 (43)	0.063
Number of household member, mean (SD)†	4.25 (1.68)	4.50 (1.77)	4.00 (1.55)	0.020
Number of children				
*None or one*	234 (81.25)	122 (84.72)	112 (77.78)	0.199
*Two*	45 (15.63)	17 (11.81)	28 (19.44)	
*Three or more*	9 (3.13)	5 (3.47)	4 (2.78)	
Comorbidities and illness history				
*History of hypertension*†	9 (3.13)	8 (5.56)	1 (0.69)	0.018
*History of diabetes*	3 (1.04)	0 (0.00)	3 (2.08)	0.082
*History of heart disease*	3 (1.04)	0 (0.00)	3 (2.08)	0.082
*History of abortion*†	53 (18.40)	33 (22.92)	20 (13.89)	0.048
*History of stillbirth*	8 (2.78)	6 (4.17)	2 (1.39)	0.151
*History of eclampsia*	4 (1.4)	2 (1.4)	2 (1.4)	1.000
*History of gestational diabetes*	2 (0.69)	2 (1.39)	0 (0.00)	0.156
Daily meal intake				
*<3 times*†	24 (8.33)	16 (11.11)	8 (5.56)	<0.001
*3 times*	191 (66.32)	113 (78.47)	78 (54.17)	
*>3 times*	73 (25.35)	15 (10.42)	58 (40.28)	
Smoking and alcohol consumption				
*Smoking of women†*	17 (5.90)	2 (1.39)	15 (10.42)	0.001
*Smoking of husband*	82 (28.50)	44 (30.56)	37 (25.69)	0.359
*Drinking of alcohol of husband*	6 (2.08)	3 (2.08)	3 (2.08)	1.000
**Clinical characteristics**				
Pregnancy duration in weeks, mean (SD)	9.83 (2.45)	9.99 (2.46)	9.66 (2.44)	0.845
Weight in kg, mean (SD)	54.63 (10.14)	52.46 (9.86)	56.29 (9.54)	<0.001
Height in cm, mean (SD)	152.77 (6.59)	153.01 (6.19)	152.54 (6.98)	0.439
Body mass index (BMI) in kg/m^2^				
*Underweight (<18.5)*†	28 (9.72)	22 (15.28)	6 (4.17)	<0.001
*Healthy weight (18.5–24.9)*	144 (50.00)	80 (55.56)	64 (44.44)	
*Overweight (25.0–29.9)*	85 (29.51)	35 (24.31)	50 (34.72)	
*Obese (≥30)*	31 (10.76)	7 (4.86)	24 (16.67)	
Blood pressure in mmHg				
*Normal (<120 and <80 mm Hg)*†	170 (59.03)	83 (57.64)	87 (60.42)	<0.001
*Elevated blood pressure ((120–129) and <80 mm Hg or both)*	52 (18.06)	38 (26.39)	14 (9.72)	
*Stage 1 hypertension ((130–139) or (80–89) mm Hg or both)*	54 (18.75)	11 (7.64)	43 (29.86)	
*Stage 2 non-severe hypertension ((140–159) or (90–109) mmHg or both)*	12 (4.17)	12 (8.33)	0 (0.00)	
Nausea or vomiting	232 (80.56)	118 (81.94)	114 (79.17)	0.551
Pedal swelling†	5 (1.74)	0 (0.00)	5 (3.47)	0.024
Vaginal bleeding (haemorrhage)	10 (3.47)	5 (3.47)	5 (3.47)	1.000

SD – standard deviation

*Values are n (%) unless specified otherwise.

†χ^2^ test or Mann-Whitney U test.

### Clinical outcomes

#### Primary outcome

A total of 183 deliveries occurred during the study period, of which 92 (50.27%) were in the IG and 91 (49.73%) were in the CG. Out of these, 77 were normal deliveries – 50 (64.94%) in the IG and the 27 (35.06%) in the CG (*P* = 0.001). C-sections were required in 106 women, of which 42 (39.62%) were in the IG and the 64 (60.38%) in the CG (*P* = 0.001). Out of the total 106 C-sections, 36 (34%) were regarded unnecessary, of which 9 (25%) occurred in the GI and 27 (75%) in the CG (*P* = 0.027) (**Table 2**). In the IG, 9/42 (21.43%) C-sections were considered unnecessary compared to 27/64 (42.19%) in the CG (*P* = 0.027) **(****Table 2****)**.

**Table 2:** Comparison of primary outcome between intervention and comparison group (n = 288)

Variables	Total	Intervention group, n (%)	Comparison group, n (%)	*P*-value
Total in each group	288	144	144	
Total completed study	183	92 (50.27)	91 (49.73)	0.903
Total number requiring C-section	106	42 (39.62)	64 (60.38)	0.001
Necessary C-section	70	33 (47.14)	37 (52.86)	0.027
Unnecessary C-section	36	09 (25)	27 (75)	
Vaginal delivery	77	50 (64.94)	27 (35.06)	0.001

C-section – caesarean section

*Pearson’s χ^2^ test.

We observed an aRR of 20.8% (95% CI = 3.3%–38.1%; *P* = 0.019) in the rate of unnecessary C-sections for the intervention group compared to the comparison group (**Table 3**). As we defined a clinically meaningful reduction as at least a 13% reduction in unnecessary C-sections, the intervention may be considered clinically meaningful.

**Table 3:** Proportion of unnecessary C-sections between intervention and comparison group (n = 288)

n (Col%)	Intervention group (n = 42), n (%)	Control group (n = 64), n (%)	aRR (95% CI)	*P*-value
Unnecessary	9 (21.4)	27 (42.2)	20.8% (3.3–38.1)	0.019
Necessary	33 (78.6)	37 (57.8)		

aRR – absolute risk reduction, CI – confidence interval, CoI% – column percentage for the proportions of intervention and control arms

#### Secondary outcomes

The average ANC visits by the women in the IG were significantly greater than those in the CG (x̄ = 4.1, SD = 2.01 *vs*. x̄ = 3.25, SD = 1.2; *P* < 0.001). On average, 51.22% of the IG group made the targeted 8 ANC visits (8 visits × 144 = 1152) and 81.25% of the CG women made 4 ANC visits (4 visits × 144 = 576). Overall, 249 (62.25%) women in IG 121 (60.5%) in the CG performed their targeted 4 and 2 USGs, respectively, out of a total of 370 USG performed by all women. There was no significant difference in institutional delivery in IG or CG (84.78% *vs*. 89.01%; *P* = 0.397). Delivery complications occurred in two cases and stillbirth in one case in the CG, while no such events occurred in the IG.

There were 1058 ANC visits – 590 in the IG and 468 in the CG. Overall, 59.03% of women in the IG and 68.05% of women in the CG attended four ANC visits. While 2.78% performed eight ANC visits in the IG. There were 370 USGs performed overall: 249 in the IG and 121 in the CG. Moreover, 15.28% women in the IG and 31.25% in the CG did not receive a USG at all, while 54.17% in the IG and 15.28% in the CG received two USGs (Table S5 in the **Online Supplementary Document**).

We performed log-linear binomial regression using a generalised linear model and found that the IG had a significantly lower risk ratio RR of 0.64 (95% CI = 0.54–0.77, *P* < 0.001) compared to the CG. Additionally, age was associated with a reduced risk of unnecessary C-sections (RR = 0.95; 95% CI = 0.93–0.98, *P* < 0.001), while BMI (RR = 1.04; 95% CI = 1.02–1.07, *P* = 0.002) and mother’s education (RR = 1.03; 95% CI = 1.00–1.06, *P* = 0.023) were linked to an increased risk independently associated with the unnecessary C-section (Table S6 in the **Online Supplementary Document**).

## DISCUSSION

We examined if unnecessary C-sections could be effectively reduced by implementing the WHO-recommended eight ANC visits along with additional USG and health education among pregnant women. We noted a significant reduction in this outcome and observed that unnecessary C-sections decreased with the mothers’ age and increased with their BMI and education.

We found that the number of ANC visits, USG uses, and normal deliveries increased significantly in the IG. Additionally, delivery-related complications such as post-partum haemorrhage and stillbirth decreased after the intervention, although this effect was not statistically significant.

In Bangladesh, four ANC visits and two USGs are recommended across the health system. Here, in our IG, we introduced four additional ANC visits to pregnant women attending health facilities, as recommended by the WHO, which we supplemented with health education delivered to the women eight times during their pregnancy and two additional USGs. All these study interventions were intended to improve the pregnant women’s knowledge and understanding regarding unnecessary C-sections and improve their confidence in normal delivery. We observed that the study interventions increased the number of ANC visits and use of USGs, resulting in increased normal delivery and reduced delivery-related complications such as post-partum haemorrhage and stillbirth. The interventions significantly reduced the overall number of C-sections and, more importantly, the number of unnecessary C-sections specifically.

We also intended to examine adherence to WHO-recommended eight times ANC visits and additional USG exams, and the feasibility of its implementation in the health system of Bangladesh. We found that the mean number of ANC visits increased in the IG, and we noted poor compliance with the scheduled additional visits. Despite the increase in the number of total visits among the IG women, we noted reduced attendance compared to the CG, particularly for the seventh visit that corresponds to the fourth visit in the CG (20.83% *vs*. 70.83%; *P* < 0.001). In IG, only seven (4.86%) pregnant women attended their eighth ANC visit and only four (2.8%) completed all eight visits (Table S7 in the **Online Supplementary Document**). We do not have a ready explanation for this observation, but a quick succession of visits, even with a two-week interval between the subsequent follow-up visits in the IG, requires planning and financial expenses (transportation cost), which could be important in the context of Bangladesh. Moreover, we hypothesise that, since we conducted our study during the COVID-19 pandemic, this might have reduced adherence to ANC visits.

Our intervention included the provision of two additional USGs (for a total of four). While the average number of USG examinations in the IG was 2.1 times higher than the average 0.8 examinations in the CG women, it is notable that 15.28% of women in the IG still never underwent USG exams throughout their pregnancy period, and that the proportion was twice higher (31.25%) among the CG. Only 40 (27.78%) women in the IG underwent USGs three times and only nine (6.25%) did so four times. We did not provide any financial incentives for their additional ANC visits and the USGs, which also might have negatively impacted adherence.

Our study involved face-to-face interviews and follow-up measurements (height, weight, examine the condition of the mother and baby) requiring the physical presence of the mothers at the hospitals. As 93.75% of our pregnant women were housewives, they mostly financially depended on their husbands and family members for financing their routine and additional ANC visits (accompaniment, transportation, hospital visits, laboratory tests, food, etc.) and their routine laboratory tests and additional USGs. This might have thus been unattainable or unaffordable for some of the women. Increasing awareness of the pregnant women’s family members and providing health education about the importance of ANC visits and USG can enhance utilisation of healthcare facilities. Further, governments, policymakers, healthcare providers, and community leaders should make ANC services accessible and available at the community level, engage community people to promote ANC, and address cultural barriers. These women also might require transportation assistance if they have difficulty reaching healthcare facilities. Healthcare providers need to build a strong rapport with the women and remind them of follow-up visits *via* phone calls and text messages. The stakeholders mentioned above should also strive to identify barriers faced by women and create a supportive environment that encourages expected mothers to prioritise ANC visits.

Health education was provided eight times to the women in the IG during their ANC visits. The WHO recommended eight ANC visits that also incorporated health education, with the aim of reducing unnecessary C-sections. This non-clinical intervention is aimed at improving pregnant women’s awareness and understanding of the potential adverse effects of a C-section, alleviate their fear, and increase their confidence in normal labour and vaginal delivery [4]. One study reported that, for pregnant women, health education, interaction with health professionals about birth, and emotional support can influence rates of unnecessary C-sections [27]. Other research from Iran reported health education to be effective in promoting normal delivery by reducing stress and pain – specifically, providing emotional and spiritual support was effective in providing them with a pleasant and satisfactory experience of a normal vaginal delivery [28].

Concerning place of delivery, there were no significant differences in institutional delivery between the IG (84.78%) and CG (89.01%), although the overall rates were much higher compared to the national 53.4% in Bangladesh in 2019 [29]. A study in Ethiopia reported that increased ANC visits resulted in increased numbers of institutional delivery (86.4%) and improvement in the women’s pregnancy-related knowledge, including in their knowledge on recognising danger signs during pregnancy [30]. We conducted our study in the referral hospitals of Bangladesh and thus it is possible that more serious cases might have attended these hospitals to avoid pregnancy-related complications.

Public hospitals are major providers of pregnancy-related services and are attended in greater numbers by economically disadvantaged individuals. We thus conducted our study in government-funded public hospitals to ensure that we gained an insight into the real-life situation with the health system of Bangladesh. Yet we note that these hospitals lack beds and are unable to admit all pregnant women for the management of any pregnancy-related complications and safe delivery of their babies, meaning that some of women might not had the opportunity to get free beds for their safe delivery in their respective hospitals, even in cases of emergency. We provided health education only to pregnant women (not their family members) who are often are not empowered to make their decisions about the choice of delivery places in our society, whereby these choices are mostly made by their husbands and in-laws. All these factors might have influenced our participants’ attendance of ANC visits.

The rate of institutional delivery was similar between IG and CG; however, the rate of normal delivery was almost double (64.94% *vs*. 35.06%) in the IG compared to the CG. This finding suggested that our combined intervention was effective in making IG women more prepared and sufficiently empowered for their normal delivery.

The number of abortions was higher in our IG (Table S8 in the **Online Supplementary Document**). In our participants, a significant proportion had elevated blood pressure and stage 2 non-severe hypertension IG: both could therefore be a risk factor for spontaneous abortion. We explored further reasons and noted that most of the abortions occurred within 20 weeks of pregnancy, *i.e.* before the second ANC visit by which women had received only one session of health education. We also noted that these women had a history of significantly higher abortions in their previous pregnancies. By providing our intervention, we might reduce postpartum haemorrhage and stillbirth among pregnant women.

### Strengths and limitations

We conducted our study at two tertiary and two district hospitals both in urban and district-level hospitals within Bangladesh’s health system, where C-sections are done routinely. Therefore, our study findings help us understand the risk factors for unnecessary C-sections in a way that could inform policymakers in designing possible solutions for the whole country.

We conducted randomisation at the hospital level rather than the individual level to prevent the possible contamination of interventions. Therefore, the stable unit treatment value assumption might not be violated. This cluster design was chosen to minimise selection bias between study groups within the same facility, as well as to minimise confusion and disruption among participants as the intervention arm would receive additional care.

As we conducted our study during the COVID-19 pandemic, this might explain the suboptimal number of ANC visits in both groups, as pregnant mothers might have been reluctant to attend the hospitals for their routine check-ups. Simultaneously, the government-funded health service providers were vastly unprepared and ill-equipped for providing routine medical care during the pandemic [31]. However, this phenomenon likely affected both study arms in the same way, meaning that it likely had no impact on our findings. In addition to this, the pandemic and the related government-enforced lockdowns might have forced women to deliver in local hospitals and clinics close to their locations, who otherwise would have delivered at one of our study sites.

 We purposefully selected two tertiary and two non-tertiary hospitals for this study. This approach makes the generalisability of the efficacy assessment limited because of the introduced selection bias and the fact that the clinical setting and practice differences, as well as the demographics and characteristics of the participants, likely differ from those of other hospitals. Nevertheless, these specific hospitals were representative of the broader national context as these are the main public tertiary and non-tertiary (district-level) hospitals in Bangladesh that serve diverse populations. We also ensured that the intervention and control arms were stratified across these different hospital types. We further acknowledge that, since we did not use a self-administered questionnaire (*i.e.* hospital staff collected the data), social desirability bias may have influenced responses, particularly due to the sensitive nature of some study topics. To minimise this, we assured participants of the anonymity of their data and that our results would be summarised at the group, rather than the individual level.

Otherwise, we minimised risks of misclassification by training our data collectors. Due to time constraints, we did not perform formative research (feasibility with the qualitative assessment) to evaluate the participants' knowledge retention or behaviour changes. We did not involve gynaecologists, obstetricians, and communities for their leadership and advocacy, as recommended by WHO, although their role might influence the rates of unnecessary C-sections in practice. However, we relied heavily on documentation in discharge sheets, which might have been prone to errors. To address this, we ensured that our operational definitions for unnecessary C-sections were adhered to across all sites to address this error. This study was an open-label intervention, which also might have led to biases. We found that the distribution of many baseline variables differed between the study groups such as women’s age, education levels of both women and their husbands, family income, history of hypertension, history of abortion, BMI, blood pressure, and pedal oedema. Although we attempted to allocate the hospitals equally as referral hospitals, pregnant women often came from various locations, including other districts. This variability may have introduced potential sources of imbalance in the data set, which likely influenced our results to some unknown extent.

## CONCLUSIONS

Our findings suggest that unnecessary C-sections could be substantially reduced by providing health education, increasing ANC visits, and performing USGs within Bangladeshi hospitals. Availability of health education materials, training staff to conduct USGs, developing and growing human resources, and increasing ANC services for safe delivery could reduce unnecessary C-sections. Ultimately, we can prevent unnecessary C-section-related complications and reduce hospital stays, hospital and patient expenses, and even maternal and child death. Integrating different policies and practices and involving all stakeholders (gynaecologists, obstetricians, anaesthesiologists, pregnant women and their family members, and policymakers) across hospitals and national context, resolving issues related to the legal and social aspects, and improving women’s attitudes towards pregnancy and childbirth are key challenges to these issues. Further interventional studies with larger sample sizes should be conducted, addressing all limitations observed in our study and incorporating primary healthcare facilities for pregnant women to determine the underlying factors associated with unnecessary C-sections. This would, in the long term, lead to further improvements in ANC visits and subsequently minimise the unnecessary C-sections.

## Additional material


Online Supplementary Document
