# Supplementary material for: Effectiveness of WHO-recommended antenatal care visits, ultrasonography, and health education in reducing unnecessary caesarean sections among pregnant women in Bangladesh: a hospital-based randomised controlled trial
Source: J Glob Health. 2025 Jun 27;15:04182. doi: 10.7189/jogh.15.04182 (PMC12204052; doi:10.7189/jogh.15.04182)
Supplement: Online Supplementary Document [file jogh-15-04182-s001.pdf]

Supplement to: Shirin H, Huq KATME, Hawlader MDH, Masud SB, Misty K, Dewan F, Moriyama M. Effectiveness of WHO-recommended antenatal care visits, ultrasonography and health education to reduce unnecessary caesarean section among pregnant women in Bangladesh: a hospital-based randomised controlled trial. J Glob Health. 2025;15:04182.

Table S1. Hospital-wise distribution of the cause of dropouts of the participants (n = 288

**Variables**	**SSMC**	**MDH**	**DMCH**	**BDH**	**Total**
Abortion	5	6	7	1	19
Stillbirth (after 20 weeks)				1	1
Withdraw consent	1	9	18	0	28
Lost to follow up	21	10	4	22	57
Other cause					
Total number	27	25	29	24	105

SSMC – Sir Salimullah Medical College Mitford Hospital, MDH – Munshiganj 250-bed District Hospital, DMCH – Dhaka Medical College Hospital, BDH – Bogra 250-bed Mohammad Ali District Hospital

Table S2. Hospital-wise distribution of the C-sections of the participants (n = 288)

**Variables**	**SSMC (IG) n=72 (%)**	**MDH (IG) n=72 (%)**	**DMCH (CG) n=72 (%)**	**BDH (CG) n=72 (%)**
**Necessary**	12 (16.67)	21 (29.17)	17 (23.61)	20 (27.78)
**Unnecessary**	5 (6.94)	4 (5.6)	11 (15.28)	16 (22.22)
**Total**	17 (23.61)	25 (34.72)	28 (38.89)	36 (50.0)

SSMC – Sir Salimullah Medical College Mitford Hospital, MDH – Munshiganj 250-bed District Hospital, DMCH – Dhaka Medical College Hospital, BDH – Bogra 250-bed Mohammad Ali District Hospital

Table S3. Baseline characteristics based on the location of the hospital (n = 288)

**Variables**		**Location**		
		**Tertiary**	**Non-tertiary**	**Total**
		**n=144**	**n=144**	**n=288**
Group	Comparison	72 (50)	72 (50)	144 (50)
	Intervention	72 (50)	72 (50)	144 (50)
Delivery	Normal	43 (48.9)	34 (35.8)	77 (42.1)
	Caesarian	45 (51.1)	61 (64.2)	106 (57.9)
C-section necessity	Necessary	29 (64.4)	41 (67.2)	70 (66)
	Unnecessary	16 (35.6)	20 (32.8)	36 (34)
Hospital	SSMCH	72 (50)	-	72 (25)
	DMCH	72 (50)	-	72 (25)
	Munshiganj GH	-	72 (50)	72 (25)
	Bogra DH	-	72 (50)	72 (25)
Age	Mean±SD	24±5	24±5	24±5
Education	Mean±SD	9±4	9±4	9±4
Age_of_husband	Mean±SD	31±6	31±6	31±6
Education_husband	Mean±SD	9±5	9±5	9±5
Income (USD/month)	Mean±SD	176±91	171±94	174±92

Expense (USD/month)	Mean±SD	131±47	140±75	136±63
Household size	Mean±SD	4±2	4±2	4±2
Meal number	Mean±SD	3±0	3±1	3±1
Religion	Muslim	139 (96.5)	135 (93.8)	274 (95.1)
	Hindu	5 (3.5)	9 (6.3)	14 (4.9)
Occupation self	Housewife*	128 (88.9)	142 (98.6)	270 (93.8)
	Others	16 (11.1)	2 (1.4)	18 (6.3)
Occupation husband	Others	28 (19.4)	36 (25)	64 (22.2)
	Industry, office non, petty	45 (31.3)	50 (34.7)	95 (33)
	Skilled, office exec., big business, overseas	71 (49.3)	58 (40.3)	129 (44.8)
Number of Children	One or no	121 (84)	113 (78.5)	234 (81.3)
	Two	19 (13.2)	26 (18.1)	45 (15.6)
	Three or more	4 (2.8)	5 (3.5)	9 (3.1)
Hypertension	Yes	4 (2.8)	5 (3.5)	9 (3.1)
Diabetes	Yes	0 (0)	3 (2.1)	3 (1)
Heart diseases	Yes	0 (0)	3 (2.1)	3 (1)
History of abortion	Yes	22 (15.3)	31 (21.5)	53 (18.4)
History of stillbirth	Yes	5 (3.5)	3 (2.1)	8 (2.8)

History of eclampsia	Yes	1 (0.7)	3 (2.1)	4 (1.4)
Meal number	< 3 times*	1 (0.7)	23 (16)	24 (8.3)
	3 times	107 (74.3)	84 (58.3)	191 (66.3)
	> 3 times	36 (25)	37 (25.7)	73 (25.3)
Smoke	Yes*	0 (0)	17 (11.8)	17 (5.9)
Alcohol	Yes	0 (0)	0 (0)	0 (0)
Smoke husband	Yes	34 (23.6)	48 (33.3)	82 (28.5)
Alcohol husband	Yes	3 (2.1)	3 (2.1)	6 (2.1)

chi-square test, Mann-Whitney U test

SD – standard deviation

**P*-value <0.05.

Table S4. Baseline characteristics based on caesarean section necessity (n = 106)

**Variables**	**Categories**	**C-section necessity**		
		**Necessary**	**Unnecessary**	**Total**
		**n=70**	**n=36**	**n=106**
Groups*	Comparison	37 (52.9)	27 (75)	64 (60.4)
	Intervention	33 (47.1)	9 (25)	42 (39.6)
Location	Tertiary	29 (41.4)	16 (44.4)	45 (42.5)
	Non-tertiary	41 (58.6)	20 (55.6)	61 (57.5)
Hospital	SSMCH	12 (17.1)	5 (13.9)	17 (16)
————	Munshiganj GH	21 (30)	4 (11.1)	25 (23.6)
	DMCH	17 (24.3)	11 (30.6)	28 (26.4)
	Bogora DH	20 (28.6)	16 (44.4)	36 (34)
Age	Mean±SD	24±5	24±4	24±5
Education	Mean±SD	10±4	10±4	10±4
Age of husband	Mean±SD	31±6	30±5	31±6
Education of husband	Mean±SD	10±5	11±5	10±5
Income (USD/month)	Mean±SD	180±89	178±93	179±90
Expense (USD/month)	Mean±SD	133±49	132±43	133±47
HH size	Mean±SD	4±2	4±2	4±2

Religion	Muslim	64 (91.4)	35 (97.2)	99 (93.4)
	Hindu	6 (8.6)	1 (2.8)	7 (6.6)
Occupation (mother)	Housewife	62 (88.6)	33 (91.7)	95 (89.6)
	Others	8 (11.4)	3 (8.3)	11 (10.4)
Occupation husband	Others	17 (24.3)	7 (19.4)	24 (22.6)
	Industry, office non, petty	24 (34.3)	9 (25)	33 (31.1)
	Skilled, office exec., big business, overseas	29 (41.4)	20 (55.6)	49 (46.2)
Number of children	One or no	56 (80)	32 (88.9)	88 (83)
	Two	12 (17.1)	3 (8.3)	15 (14.2)
	Three or more	2 (2.9)	1 (2.8)	3 (2.8)
Hypertension	Yes	3 (4.3)	1 (2.8)	4 (3.8)
Diabetes	Yes	0 (0)	1 (2.8)	1 (0.9)
Heart diseases	Yes	2 (2.9)	0 (0)	2 (1.9)
History of haemorrhage	Yes	0	2 (5.6)	2 (1.9)
History of abortion	Yes	10 (14.3)	10 (27.8)	20 (18.9)
History of stillbirth	Yes	1 (1.4)	0 (0)	1 (0.9)
History of eclampsia	Yes	1 (1.4)	1 (2.8)	2 (1.9)
Meal number	< 3 times	8 (11.4)	3 (8.3)	11 (10.4)

	3 times	37 (52.9)	19 (52.8)	56 (52.8)
	> 3 times	25 (35.7)	14 (38.9)	39 (36.8)
Smoking of mother	Yes	4 (5.7)	5 (13.9)	9 (8.5)
Alcohol	Yes	0 (0)	0 (0)	0 (0)
Smoking of father	Yes	16 (22.9)	8 (22.2)	24 (22.6)
Alcohol consumption by husband	Yes	3 (4.3)	0 (0)	3 (2.8)

Chi-square test, Mann-Whitney U test

SD – standard deviation

**P*=0.003

Table S5. Comparison of secondary outcomes between intervention and comparison group (n = 288)

Variables	Intervention group n (%)	Comparison group n (%)	*P*-value
**ANC**	8 times Success /Fail	4 times Success/Fail	
Visit 1 (8-12w)	144 (100%)	144 (100%)	-
Visit 2 (20w)	123 (85.42%)		
Visit 3 (24-26w)	87 (60.42%)	114 (79.17%)	0.001
Visit 4 (30w)	76 (52.78%)		
Visit 5 (34w)	72 (50.00%)	108 (75.00%)	<0.001
Visit 6 (36w)	51 (35.42%)		
Visit 7 (38w)	30 (20.83%)	102 (70.83%)	<0.001
Visit 8 (40w)	7 (4.86%)		
Sum of all individual ANC visits	590/1152 (51.22%)	468/576 (81.25%)	
Individual ANC visits (total)	590/1058 (55.77%)	468/1058 (44.23%)	<0.001
ANC (mean±SD)/ person	4.1±2.01	3.25±1.2	<0.001
**PNC**	92 (63.89%)	91 (63.19%)	0.903
**USG**			
USG 1 (8-12w)	85/144 (59.03%)	28/144 (19.44%)	<0.001
USG 2 (24-26w)	75/144 (52.08%)		
USG 3 (34w)	60/144 (41.67%)		
USG 4 (38w)	29/144 (20.14%)	93/144 (64.58%)	<0.001
Total USG by all participants	249/4 (62.25%)	121/2 (60.5%)	
Average USG	1.73 (249/144)	0.84 (121/144)	<0.001
Individual USG	249 (67.3%; 249/370)	121 (32.7%; 121/370)	
**Delivery**			
Institutional delivery	78/92 (84.78%)	81/91 (89.01%)	0.397
Home delivery	14/92 (15.22%)	10/91 (10.99%)	
**Complications**			
Delivery complication (PPH)	0	2	0.345
Stillbirth	0	1	0.316

N – number, % – percentage

P-value <0.05; USG – Ultrasonography

Table S6. Results of log-linear binomial regression models, both adjusted and unadjusted, indicating the associated factors for unnecessary C-section delivery

Variables (type)	Unadjusted		Adjusted	
	**cRR (95% CI)**	**p-value**	**aRR (95% CI)**	***P*-value**
Groups				
Comparison	Ref.		Ref.	
Intervention	0.65 (0.47 – 0.90)	0.009	0.64 (0.54 – 0.77)	<0.001
Location				
Tertiary	Ref.		Ref.	
Non-tertiary	1.33 (0.82 – 2.17)	0.247	1.39 (1.24 – 1.57)	<0.001
Age of the mother	0.98 (0.95 – 0.99)	0.029	0.95 (0.93 – 0.98)	<0.001
BMI of Mothers	1.03 (1.00 – 1.07)	0.040	1.04 (1.02 – 1.07)	0.002
Mothers years of education	1.04 (1.00 – 1.00)	0.043	1.03 (1.00 – 1.06)	0.023
Mothers occupation				
Housewife	Ref.		Ref.	
Others	2.80 (0.92 – 3.50)	0.084	2.7 (2.04 – 3.57)	<0.001
Mother's smoking habit				
No	Ref.		Ref.	
Yes	1.45 (1.04 – 2.00)	0.025	1.33 (0.96 – 1.85)	0.088
Presence of comorbidities*				

No	Ref.		Ref.	
Yes	1.15 (0.85 – 1.56)	0.358	1.56 (1.14 – 2.15)	0.006

cRR – crude risk ratio, aRR – adjusted risk ratio, Cl – confidence interval

*Comorbidities: hypertension, diabetes and heart diseases.

Table S7. ANC visits and USG performed by the individual in IG and CG (n = 288)

Number of ANC visits	Total ANC n (%)	IG n (%)	CG n (%)
1 ANC Visit	288	144	144
2 ANC Visit	244 (84.72)	127 (88.19)	11 7 (81.25)
3 ANC Visit	212 (73.61)	103 (71.53)	109 (75.69)
4 ANC Visit	183 (63.54)	85 (59.03)	98 (68.05)
5 ANC Visit	65 (22.57)	65 (45.14)	
6 ANC Visit	45 (15.62)	45 (31.25)	
7 ANC Visit	17 (5.90)	17 (11.80)	
8 ANC Visit	4 (1.39)	4 (2.78)	
**Total ANC visits**	**1058**	**590**	468
**Number of USG**			
0	67 (23.26)	22 (15.28)	45 (31.25)
1	221 (76.74)	122 (84.72)	99 (68.75)
2	100 (34.72)	78 (54.17)	22 (15.28)
3	40 (13.89)	40 (27.78)	
4	9 (3.12)	9 (6.25)	
**Total number**	**370**	**249**	**121**

ANC – antenatal care, n – number, % – percentage, IG – intervention group, CG – comparison group, USG – ultrasonography

Table S8. Distribution of cause of dropout/completion in different visits of both intervention and comparison groups (n = 288)

Group	Drop out/ completed	Cause of dropout	ANC visits								PNC	Total
			1	2	3	4	5	6	7	8		
			8-12 wks	20 wks	26 wks	30 wks	34	36	38	40	PNC	
Intervention Group (n=144)	Drop out /total			17	19	6	5	2	1	2		52
		Withdraw			6	1		1		2		10
		Lost to follow-up		12	8	4	5	1	1			31
		Abortion		5	5	1						11
		Stillbirth										0
	Follow-up completed/total											92
Comparison Group (n=144)	Drop out /total		8-12 wks		26 wks		34 wks		38 wks		PNC	53
		Withdraw			1						17	18
		Lost to follow-up			16		3		5		2	26
		Abortion			8							8
		Stillbirth									1	1
	Follow-up completed/total											91

ANC – antenatal care, PNC – postnatal care, wks – weeks

Checklist S1. CONSORT checklist for study

CONSORT 2010 checklist of information to include when reporting a randomised trial*

Section/Topic	Item No	Checklist item	Reported on page No
**Title and abstract**			
	1a	Identification as a randomised trial in the title	1
	1b	Structured summary of trial design, methods, results, and conclusions (for specific guidance see CONSORT for abstracts)	1-2
**Introduction**			
Background and objectives	2a	Scientific background and explanation of rationale	2-5
	2b	Specific objectives or hypotheses	5-6
**Methods**			
Trial design	3a	Description of trial design (such as parallel, factorial) including allocation ratio	6
	3b	Important changes to methods after trial commencement (such as eligibility criteria), with reasons	N/A
Participants	4a	Eligibility criteria for participants	7-8
	4b	Settings and locations where the data were collected	6-7
Interventions	5	The interventions for each group with sufficient details to allow replication, including how and when they were actually administered	8-9
Outcomes	6a	Completely defined pre-specified primary and secondary outcome measures, including how and when they were assessed	12
	6b	Any changes to trial outcomes after the trial commenced, with reasons	N/A

Sample size	7a	How sample size was determined	12-13
	7b	When applicable, explanation of any interim analyses and stopping guidelines	N/A
Randomisation:			
Sequence generation	8a	Method used to generate the random allocation sequence	7
	8b	Type of randomisation; details of any restriction (such as blocking and block size)	6
Allocation concealment mechanism	9	Mechanism used to implement the random allocation sequence (such as sequentially numbered containers), describing any steps taken to conceal the sequence until interventions were assigned	6-7
Implementation	10	Who generated the random allocation sequence, who enrolled participants, and who assigned participants to interventions	7-8
Blinding	11a	If done, who was blinded after assignment to interventions (for example, participants, care providers, those assessing outcomes) and how	7
	11b	If relevant, description of the similarity of interventions	6
Statistical methods	12a	Statistical methods used to compare groups for primary and secondary outcomes	13
	12b	Methods for additional analyses, such as subgroup analyses and adjusted analyses	13
**Results**			
Participant flow (a diagram is strongly recommended)	13a	For each group, the numbers of participants who were randomly assigned, received intended treatment, and were analysed for the primary outcome	27
	13b	For each group, losses and exclusions after randomisation, together with reasons	27
Recruitment	14a	Dates defining the periods of recruitment and follow-up	6

	14b	Why the trial ended or was stopped	N/A
Baseline data	15	A table showing baseline demographic and clinical characteristics for each group	28-29
Numbers analysed	16	For each group, number of participants (denominator) included in each analysis and whether the analysis was by original assigned groups	14
Outcomes and estimation	17a	For each primary and secondary outcome, results for each group, and the estimated effect size and its precision (such as 95% confidence interval)	29-30
	17b	For binary outcomes, presentation of both absolute and relative effect sizes is recommended	29
Ancillary analyses	18	Results of any other analyses performed, including subgroup analyses and adjusted analyses, distinguishing pre-specified from exploratory	30
Harms	19	All important harms or unintended effects in each group (for specific guidance see CONSORT for harms)	18
**Discussion**			
Limitations	20	Trial limitations, addressing sources of potential bias, imprecision, and, if relevant, multiplicity of analyses	21-22
Generalisability	21	Generalisability (external validity, applicability) of the trial findings	21
Interpretation	22	Interpretation consistent with results, balancing benefits and harms, and considering other relevant evidence	15-16
**Other information**			
Registration	23	Registration number and name of trial registry	23
Protocol	24	Where the full trial protocol can be accessed, if available	23
Funding	25	Sources of funding and other support (such as supply of drugs), role of funders	24

Citation: Schulz KF, Altman DG, Moher D, for the CONSORT Group. CONSORT 2010 Statement: updated guidelines for reporting parallel group randomised trials. BMC Medicine. 2010;8:18.
© 2010 Schulz et al. This is an Open Access article distributed under the terms of the Creative Commons Attribution License

(http://creativecommons.org/licenses/by/2.0), which permits unrestricted use, distribution, and reproduction in any medium, provided the original work is properly cited.

*We strongly recommend reading this statement in conjunction with the CONSORT 2010 Explanation and Elaboration for important clarifications on all the items. If relevant, we also recommend reading CONSORT extensions for cluster randomised trials, non-inferiority and equivalence trials, non-pharmacological treatments, herbal interventions, and pragmatic trials. Additional extensions are forthcoming: for those and for up-to-date references relevant to this checklist, see www.consort-statement.org.
